# Prevalence and associated factors of cigarette smoking and substance use among university entrance test-taking students: A GIS-based study

**DOI:** 10.1371/journal.pone.0308697

**Published:** 2024-08-22

**Authors:** Mohammed A. Mamun, Nitai Roy, David Gozal, Moneerah Mohammad Almerab, Md. Shakhaoat Hossain, Firoj Al Mamun

**Affiliations:** 1 CHINTA Research Bangladesh, Savar, Dhaka, Bangladesh; 2 Department of Public Health and Informatics, Jahangirnagar University, Savar, Dhaka, Bangladesh; 3 Department of Public Health, University of South Asia, Dhaka, Bangladesh; 4 Department of Biochemistry and Food Analysis, Patuakhali Science and Technology, Patuakhali, Bangladesh; 5 Joan C. Edwards School of Medicine, Marshall University, Huntington, WV, United States of America; 6 Department of Psychology, College of Education and Human Development, Princess Nourah Bint Abdulrahman University, Riyadh, Saudi Arabia; Bangladesh University of Health Sciences, BANGLADESH

## Abstract

**Background:**

Numerous studies have examined substance use and smoking behavior among adolescents and university students. However, little is known about these behaviors among students undergoing university entrance tests, a critical transition period from adolescence to adulthood. The entrance test can significantly affect students’ mental health, potentially leading to substance use. This study aims to investigate the prevalence of cigarette smoking and substance use among students taking these exams and the associated factors.

**Methods:**

A cross-sectional survey was carried out on September 4th and 11th, 2022 to collect data from 1,480 university entrance test-taking students using a convenience sampling technique. Chi-square tests and logistic regression were conducted using SPSS software. Besides, GIS mapping was used to visualize the distribution of substance use and smoking behavior across districts via ArcGIS.

**Results:**

The study found a 10% prevalence of current tobacco smoking and 4% substance use. Females (OR = 1.98; 95% CI: 1.38–2.85), urban residence (OR = 2.03; 95% CI: 1.42–2.88), repeater (OR = 1.45; 95% CI: 1.02–2.06), anxiety (OR = 1.55, 95% CI: 1.10–2.19), burnout (OR = 1.51, 95% CI: 1.00–2.12), and suicidal behavior (OR = 1.57; 95% CI: 1.03–2.40) were the significant factors for cigarette use. Whereas the urban residence (OR = 1.91; 95% CI: 1.11–3.31), anxiety (OR = 2.47, 95% CI: 1.45–4.20), and suicidal behavior (OR = 2.76; 95% CI: 1.55–4.92) significantly increased the risk of substance use. GIS analysis revealed males varied in substance use and females in tobacco smoking by district. Repeat test-takers were associated with district variations in both smoking and substance use.

**Conclusions:**

Educational institutions, public health authorities, and policymakers must implement mental health support and substance use prevention programs for students. Integrating mental health education, providing resources, and enforcing regulations can promote healthier coping strategies and reduce substance use risks among students.

## 1 Introduction

Substance use, encompassing behaviors like smoking, alcohol consumption, and illicit drug use, is a prevalent and concerning issue worldwide [1, 2]. This behavior often leads to dependence syndrome, posing significant negative impacts on mental and physical health as well as adverse economic consequences [3]. Recent estimates from the UN Office on Drugs and Crime indicate that over 284 million individuals aged 15–64 years report substance use, with the prevalence increasing by 26% over the past decade [4]. It has been observed that young people are increasingly likely to consume drugs, and in several countries, the level of usage is higher than in the previous generation. In Bangladesh, recent reports suggest that approximately 7.5 million people use drugs in 2020; of these, 65% are aged 15 years and above. Notably, during difficult periods such as the COVID-19 pandemic, the number of drug users increased to over 8 million people, with the majority of drug users being young people aged 15 to 35 [5].

Adolescence is a critical transitional period characterized by rapid physiological and psychosocial changes. During this phase, many adolescents adopt unhealthy behaviors, including substance use. A large proportion of substance users begin experimenting with during their teenage years, and research shows that over 90% of those who develop addictive behaviors were introduced to substance use before reaching adulthood [6]. This means that most people who struggle with addiction start drinking alcohol or substance use before they turn 18 years of age. A review of 26 studies, with 19 studies involving students transitioning to post-secondary education and seven studies examining university students directly, investigated the prevalence trends of substance use [2]. Notably, the transition from high school to higher education is a vulnerable period, as substance use tends to increase during this time, influenced by factors such as peer influence, psychological issues, and mental health concerns [2]. Understanding the predictive factors and patterns of substance use during this pivotal phase is therefore crucial for addressing its long-term consequences.

Adolescent drug abuse has been linked to various adverse outcomes, including depression, anxiety, sleep problems, low self-esteem, aggression, antisocial behavior, delinquency, crime, and rebelliousness [3, 7]. Early initiation of cigarette smoking significantly raises the risk of future nicotine dependency, and alcohol and tobacco use during adolescence may pave the way for experimenting with more serious addictive substances later in life [8]. Moreover, substance use substantially impacts academic performance, as evidenced by studies linking it to low academic achievement. For instance, a nationally representative survey in the US among 12th graders revealed that those who frequently smoked, engaged in binge drinking, or used marijuana were more likely to have lower academic performance (C grades or below) [9]. Substance users also had a higher risk of skipping school, while lifetime non-users demonstrated greater academic self-efficacy and emotional and academic engagement than past-year users [9]. In addition, academic-related stress emerged as the primary source of stress among 88.6% of students [10]. Stress was significantly associated with the following relative risk increases: khat chewing 3.03-fold, cigarette smoking 4.55-fold, and alcohol intake 1.93-fold. Furthermore, stress was significantly negatively correlated with academic achievement [10].

In Bangladesh, gaining admission to a university for tertiary education is highly competitive due to the limited seats available for many high school graduates. This intense competition creates a significant impact on student’s mental health, who often have to prepare for multiple exam syllabi and take entrance exams at various locations. Previous studies have highlighted the burdens of mental health problems experienced by students taking university entrance tests. For instance, about 47.9% of students reported depression, 28.9% experienced anxiety, and 43.7% exhibited symptoms of burnout [11, 12]. This stressful environment may contribute to increased substance use among students as a coping mechanism. Therefore, it is crucial to examine the prevalence and factors associated with cigarette smoking and substance use in this population.

In Bangladesh, previous studies reported the prevalence of smoking as 37% among university students [13]. Another study conducted among male youths aged 15–24 years in rural areas reported that the prevalence of smoking cigarettes or bidis was 40.3% [14]. Results from the International Tobacco Control Bangladesh Survey reported that 20% of adults were smokeless tobacco users, while females, illiterate individuals and those living in slum areas were at higher risk of smokeless tobacco use [15]. Another population-based household survey revealed that 23% of the respondents used any form of smokeless tobacco (10.4% used only sadapatha, 13.6% used only zarda, and 2.2% used both), with males reporting higher rates than females (27% vs. 19.3%) [16].

However, those studies did not focus on a vulnerable cohort such as university entrance test-taking students with a nationwide distribution of cigarette smoking and substance use. Therefore, the present study aimed to examine the prevalence of cigarette smoking and substance use among students taking university entrance tests, identify the factors associated with these behaviors, and employ Geographical Information System (GIS) techniques to analyze their spatial distribution, marking the first time such an approach is used in this context. Understanding the prevalence, associated factors, and spatial distribution of substance use among students taking university entrance tests can provide valuable insights into the broader impact of substance abuse on mental health and academic performance during this crucial phase of life.

## 2 Methods

### 2.1 Study participants and procedure

A cross-sectional study was conducted among students preparing to take university entrance tests. The participants were recent high school graduates, typically aged between 18 and 20 years. The study focused on students who attended the university entrance test at Jahangirnagar University between September 4th and 11th, 2022 and data were collected within this time. The research team approached students who resided in the university dormitories and provided them with detailed information about the study’s objectives and the questionnaire. Data were collected using a self-reported approach, employing a non-probability sampling technique. Initially, questionnaires were collected from 1,574 students, with a total of 1,480 responses being finally included in the analysis after excluding incomplete responses related to important variables.

### 2.2 Measures

#### 2.2.1 Sociodemographic factors

Socio-demographic information was collected from the students which included gender (male vs. female), location of permanent residence (urban vs. rural areas), family type (nuclear vs. joint), and monthly family income. The participants’ socioeconomic status was determined based on their monthly family income, categorized as poor (income less than 15,000 Bangladeshi Taka [BDT]), middle-class (income ranging between 15,000–30,000 BDT), and high-class (income more than 30,000 BDT) following previous studies [17].

#### 2.2.2 Admission test-related variables

Data pertaining to the test-taking status of the participants were collected, including whether they were first-time or repeat test takers, as well as their educational background in high school and various additional related dimensions. The participants’ Grade Point Average (GPA) in public exams was also recorded. Furthermore, the participants were asked about their preparation for the examination, such as whether they sought the assistance of tutors or coaching facilities, their performance on practice exams, their monthly expenditures during the admission test preparation, and their preferred academic institution aimed for admission.

#### 2.2.3 Patient Health Questionnaire

The depression status of the participants was assessed using the Patient Health Questionnaire (PHQ-9) [18]. The PHQ-9 consists of 9 items, and participants were asked to rate their experiences over the past two weeks using a four-point Likert scale (0 = not at all, to 3 = nearly every day). The scores range from 0 to 27, whereas a cut-off score of ≥10 was used to identify the presence of depression [18]. The scale was previously employed in the same population in Bangladesh [11, 12, 19]. The internal consistency of the PHQ-9 in this study was measured using Cronbach’s alpha (α = 0.76).

#### 2.2.4 Generalized anxiety disorder

To assess anxiety traits, we employed the Generalized Anxiety Disorder (GAD-7) scale [20]. The GAD-7 consists of 7 items, and participants were asked to rate their experiences over the past two weeks using a four-point Likert scale (0 = not at all, to 3 = nearly every day). The scores range from 0 to 21, whereas a cut-off score of ≥10 was used to identify the presence of anxiety. The scale was previously employed in the same population in Bangladesh [11, 12, 19]. The internal consistency of the GAD-7 in this study was measured using Cronbach’s alpha (α = 0.83).

#### 2.2.5 Maslach burnout inventory—Student survey

To evaluate burnout in students, the Maslach Burnout Inventory–Student Survey (MBI-SS) was used [21]. The MBI-SS includes 15 items that are responded to on a 7-point Likert scale ranging from 0 (strongly disagree) to 6 (strongly agree). The inventory consists of three subscales: exhaustion (4 items), cynicism (5 items), and efficacy (6 items). The scores on these subscales are calculated separately, with emotional exhaustion scores ranging from 0 to 24, cynicism scores from 0 to 30, and academic efficacy scores from 0 to 36. Higher scores on exhaustion (low = 0–9; moderate = 10–14; high > 14) and cynicism (low = 0–1; moderate = 2–6; high > 6), and lower scores on academic efficacy (low ≤ 22; moderate = 23–27; high ≥ 28) subscale are indicative of burnout [22]. Here, two-dimensional burnout was calculated considering higher emotional exhaustion and cynicism scores. The scale was previously employed in the same population in Bangladesh [11, 12, 19]. The internal consistency of the MBI-SS was measured by Cronbach’s alpha (α = 0.85) in the present study.

#### 2.2.6 Suicidal behaviors

By a single question, participants were asked if they had experienced at least thoughts of dying by suicide in the past year, indicating suicidal behavior. The assessment of the behavior followed a similar approach to a previous study conducted among university entrance test-taking students, using binary response options (Yes/No) [12, 19].

#### 2.2.7 Outcome variables

This study collected information on two outcome variables: cigarette smoking, and substance use by asking two questions that were adapted from a previous study conducted among Bangladeshi students [23]. In this study, substance use is operationally defined as the consumption of any psychoactive substances, including alcohol, and illicit drugs. The specific questions asked were: “Have you smoked a cigarette at least once in the past 30 days?” and “Have you used any substances (e.g., alcohol, illicit drugs) at least once in the past 30 days?” with a binary (yes/no) response.

### 2.3 Statistical analysis

Microsoft Excel 2019 was utilized to input and depurate the data, while the Statistical Package for Social Science (SPSS) version 25 was employed for statistical analyses. The study used descriptive (i.e., frequency and percentages) and inferential statistics (i.e., chi-square and logistic regression). The chi-square test was conducted to ascertain the association between cigarette use status and substance use status and the study variables. Fisher’s Exact test was used when more than 20% of the cells have expected frequencies less than 5. Binary logistic regression was performed to extract the associated factors of cigarette smoking status and substance use status. The results from binary logistic regression were presented as the odds ratio with a 95% confidence interval. ArcGIS 10.8 software was used for spatial analysis across districts in terms of cigarette use status and substance use status. The geographical distribution was cross-checked with free and open-access resources such as DIVA-GIS (https://diva-gis.org/) and Bangladesh Government mapping sites (https://oldweb.lged.gov.bd/ViewMap.aspx) to ensure accuracy and consistency. All statistical tests were considered significant at *p*<0.05, with a 95% confidence interval.

### 2.4 Ethical statement

The present study was conducted in accordance with the Declaration of Helsinki 2013. The study was approved by CHINTA Research Bangladesh (approval number: chinta/2022/a7). All of the participant’s information was anonymously presented in this study. Before including in the study, participants were required to provide written informed consent. Participation was voluntary and they had the right to decline participation or withdraw at any stage of the study.

## 3 Results

### 3.1 Description of the study participants

The study involved 1,480 students, with 78.4% being males, and most living in rural areas (73.9%). Most of the test-taking candidates were Muslim (85.8%) and originated from nuclear families (73.9%). Additionally, 71.1% of the participants were first-time test takers, and 69.0% obtained professional help to prepare for their tests (**Table 1**).

**Table 1:** Distribution of the study variables by smoking status and substance use status.

Variables	Total Sample	Cigarette Use Status			Substance Use Status		
	n; (%)	Yes; n (%)	*χ*^*2*^ test value	*p*-value	Yes; n (%)	*χ*^*2*^ test value	*p*-value
**Socio-demographic variables**							
**Gender**							
Male	1160; 78.4%	101; 8.7%	14.229	<0.001	49; 4.2%	1.327	0.249
Female	320; 21.6%	51; 15.9%			9; 2.8%		
**Permanent residence**							
Rural	1094; 73.9%	92; 8.4%	16.018	<0.001	35; 3.2%	5.625	0.018
Urban	369; 24.9%	58; 15.7%			22; 6.0%		
**Religion**							
Muslim	1270; 85.8%	126; 9.9%	0.907	0.341	49; 3.9%	0.352	0.553
Others	189; 12.8%	23; 12.2%			9; 4.8%		
**Family type**							
Nuclear	1093; 73.9%	109; 10.0%	0.111	0.738	40; 3.7%	0.721	0.396
Joint	342; 23.1%	32; 9.4%			16; 4.7%		
**Monthly income (BDT)**							
<15000	372; 25.1%	21; 5.6%	12.316	0.002	4; 1.1%	29.365	<0.001
15000–30000	377; 25.5%	38; 10.1%			13; 3.4%		
>30000	269; 18.2%	37; 13.8%			26; 9.7%		
**Admission-related variables**							
**Appearance in the admission test**							
First-time test-takers	1052; 71.1%	97; 9.2%	4.350	0.037	39; 3.7%	0.433	0.511
Repeat test-takers	428; 28.9%	55; 12.9%			19; 4.4%		
**Secondary School Certificate (SSC) grade point average**							
Poor (<4.5)	296; 20.0%	32; 10.8%	1.160	0.560	14; 4.7%	4.666	0.097
Moderate	477; 32.2%	41; 8.6%			11; 2.3%		
High (5)	537; 36.3%	54; 10.1%			25; 4.7%		
**Higher Secondary Certificate (HSC) grade point average**							
Poor (<4.5)	145; 9.8%	18; 12.4%	3.390	0.184	12; 8.3%	10.847	0.004
Moderate	380; 25.7%	42; 11.1%			8; 2.1%		
High (5)	780; 52.7%	66; 8.5%			30; 3.8%		
**Coached by professional coaching centers**							
Yes	1021; 69.0%	94; 9.2%	0.603	0.438	35; 3.4%	2.535	0.111
No	378; 25.5%	40; 10.6%			20; 5.3%		
**Desired institute/department for admission**							
University	1081; 73.0%	108; 10.0%	6.713	0.082	28; 2.6%	27.492*	<0.001
Medical School	217; 14.7%	17; 7.8%			11; 5.1%		
Engineering School	88; 5.9%	8; 9.1%			8; 9.1%		
Agricultural School	31; 2.1%	7; 22.6%			7; 22.6%		
**Satisfied with previous mock test performance**							
Yes	502; 33.9%	46; 9.2%	0.026	0.872	24; 4.8%	1.171	0.279
No	838; 56.6%	79; 9.4%			30; 3.6%		
**Average monthly expenditure during admission test (BDT)**							
<5,000	202; 13.6%	16; 7.9%	1.569	0.456	4; 2.0%	3.064	0.216
5000–10,000	594; 40.1%	59; 9.9%			20; 3.4%		
>10,000	216; 14.6%	25; 11.6%			11; 5.1%		
**Educational background**							
Science	868; 58.6%	96; 11.1%	4.416	0.110	36; 4.1%	0.233	0.890
Arts	497; 33.6%	40; 8.0%			18; 3.6%		
Commerce	104; 7.0%	14; 13.5%			4; 3.8%		
**Mental health problems**							
**Depression**							
No	667; 45.1%	58; 8.7%	3.267	0.071	20; 3.0%	2.732	0.098
Yes	813; 54.9%	94; 11.6%			38; 4.7%		
**Anxiety**							
No	976; 65.9%	86; 8.8%	6.618	0.010	26; 2.7%	11.988	<0.001
Yes	504; 34.1%	66; 13.1%			32; 6.3%		
**Burnout**							
No	958; 64.7%	85; 8.9%	5.757	0.016	36; 3.8%	0.187	0.665
Yes	522; 35.3%	67; 12.8%			22; 4.2%		
**Suicidal behavior**							
No	1263; 85.3%	121; 9.6%	4.449	0.035	40; 3.2%	12.932	<0.001
Yes	217; 14.7%	31; 14.3%			18; 8.3%		

*Fisher’s Exact test

The overall prevalence of depression and anxiety among the participants was 54.9% and 34.1%, respectively, whereas 35.3% reported burnout symptoms (**Table 1**). Of the respondents, 10.3% (n = 152) reported that they were current cigarette users, whereas 3.9% (n = 56) reported being current substance users.

### 3.2 Cigarette smoking and substance use

Female students had a significantly higher rate of cigarette smoking compared to their male counterparts (15.9% vs. 8.7), but no significant differences emerged in substance use. Students from urban areas were had a significantly higher rate of smoking cigarettes and substance use than rural students (15.7% vs. 8.4%;, and 6% vs. 3.2%, respectively) (**Table 1**).

Students who attempted the test for their second time had a higher prevalence of cigarette smoking compared to those taking the test for the first time (12.9% vs. 9.2%), but no differences emerged as far as substance use status. However, higher secondary school grades and aimed admission to the institute were significantly associated with substance use status (**Table 1**).

If anxiety was present, a significantly higher prevalence of cigarette and substance use was reported (13.1% vs. 8.8%, and 6.3% vs. 2.7%, respectively). Participants reporting burnout significantly consumed cigarettes than those without burnout (12.8% vs. 8.9%). Finally, participants having suicidal behavior were more prevalent to be cigarette and substance users, that is, 14.3% vs. 9.6% and 8.3% vs. 3.2%, respectively (**Table 1**).

### 3.3 Associated factors of the usages of cigarette and substance

In terms of gender, female participants had a 1.98 times higher risk of cigarette smoking (95% CI: 1.38–2.85), where the relationship was not significant for substance use status. However, compared with the rural ones, urban students had the risk of developing cigarette smoking 2.031 times (95% CI: 1.42–2.88) and substance use by 1.91 times (95% CI: 1.11–3.31). Regarding monthly family income, the students with higher family income reported a higher risk for cigarette and substance use. **(Table 2)**.

**Table 2:** Binary logistic regression analysis concerning smoking status and substance use status.

Variables		Cigarette Use Status				Substance Use Status			
		OR		95% CI	*p*-value	OR		95% CI	*p*-value
**Socio-demographic variables**									
**Gender**									
	Male	Reference			<0.001	Reference			0.253
	Female	1.98		1.38–2.85		0.65		0.31–1.35	
**Permanent residence**									
	Rural	Reference			<0.001	Reference			0.020
	Urban	2.03		1.42–2.88		1.91		1.11–3.31	
**Religion**									
	Muslim	Reference			0.342	Reference			0.554
	Others	1.25		0.78–2.01		1.24		0.60–2.58	
**Family type**									
	Nuclear	Reference			0.738	Reference			0.397
	Joint	0.93		0.61–1.41		1.29		0.71–2.33	
**Monthly income (BDT)**									
	<15000	Reference				Reference			
	15000–30000	1.87		1.07–3.25	0.026	3.28		1.06–10.17	0.039
	>30000	2.66		1.52–4.66	0.001	9.84		3.39–28.55	<0.001
**Admission-related variables**									
**Appearance in the admission test**									
	Fresher test-takers	Reference			0.038	Reference			0.511
	Repeat test-takers	1.45		1.02–2.06		1.20		0.68–2.11	
**Secondary School Certificate (SSC) grade point average**									
	Poor (<4.5)	Reference				Reference			
	Moderate	0.77		0.47–1.26	0.307	0.47		0.21–1.06	0.070
	High (5)	0.92		0.58–1.46	0.732	0.98		0.50–1.92	0.961
**Higher Secondary Certificate (HSC) grade point average**									
	Poor (<4.5)	1.53	0.88–2.66		0.131	2.25	1.12–4.51		0.022
	Moderate	1.34	0.89–2.02		0.155	0.53	0.24–1.18		0.124
	High (5)	Reference				Reference			
**Coached by professional coaching centers**									
	Yes	Reference			0.438	Reference			0.114
	No	1.16		0.79–1.72		1.57		0.89–2.76	
**Desired institute/department for admission**									
	Varsity	Reference				Reference			
	Medical	0.76		0.49–1.30	0.327	2.00		0.98–4.09	0.055
	Engineering	0.90		0.42–1.91	0.786	3.76		1.66–8.52	0.002
	Agriculture	2.62		1.10–6.24	0.029	10.96		4.36–27.57	<0.001
**Satisfied with previous mock tests**									
	Yes	Reference			0.872	Reference			0.281
	No	1.03		0.70–1.51		0.73		0.42–1.28	
**Average monthly expenditure (BDT)**									
	<5,000	Reference				Reference			
	5000–10,000	1.28		0.72–2.28	0.399	1.72		0.58–5.10	0.325
	>10,000	1.52		0.78–2.94	0.212	2.65		0.83–8.48	0.099
**Educational background**									
	Science	Reference				Reference			
	Arts	0.70		0.47–1.03	0.075	0.86		0.48–1.54	0.632
	Commerce	1.25		0.68–2.28	0.466	0.92		0.32–2.65	0.884
**Mental health problems**									
**Depression**									
	No	Reference			0.072	Reference			0.101
	Yes	1.37		0.97–1.93		1.58		0.91–2.75	
**Anxiety**									
	No	Reference			0.011	Reference			<0.001
	Yes	1.55		1.10–2.19		2.47		1.45–4.20	
**Burnout**									
	No	Reference			0.017	Reference			0.665
	Yes	1.51		1.00–2.12		1.12		0.65–1.93	
**Suicidal behavior**									
	No	Reference			0.036	Reference			<0.001
	Yes	1.57		1.03–2.40		2.76		1.55–4.92	

Compared with the fresher test-takers, 1.45 times more likely to have the risk of cigarette use among repeat test-taking students (95% CI: 1.02–2.06), but there is no difference in factors associated with the student status and substance use. Anxious students were more likely to be cigarette and substance users by 1.55 times (95% CI: 1.10–2.19) and 2.47 times (95% CI: 1.45–4.20), respectively. In contrast, burnout students were reported to be at an increased risk of 1.51 times (95% CI: 1.00–2.12) for cigarette smoking. Finally, students experiencing suicidal behavior had 1.57 times (95% CI: 1.03–2.40) and 2.76 times (95% CI: 1.55–4.92) more risk of smoking cigarettes and using substances than those who had not (**Table 2**).

### 3.4 GIS-based distribution of the usages of cigarettes and substance

**Fig 1:**
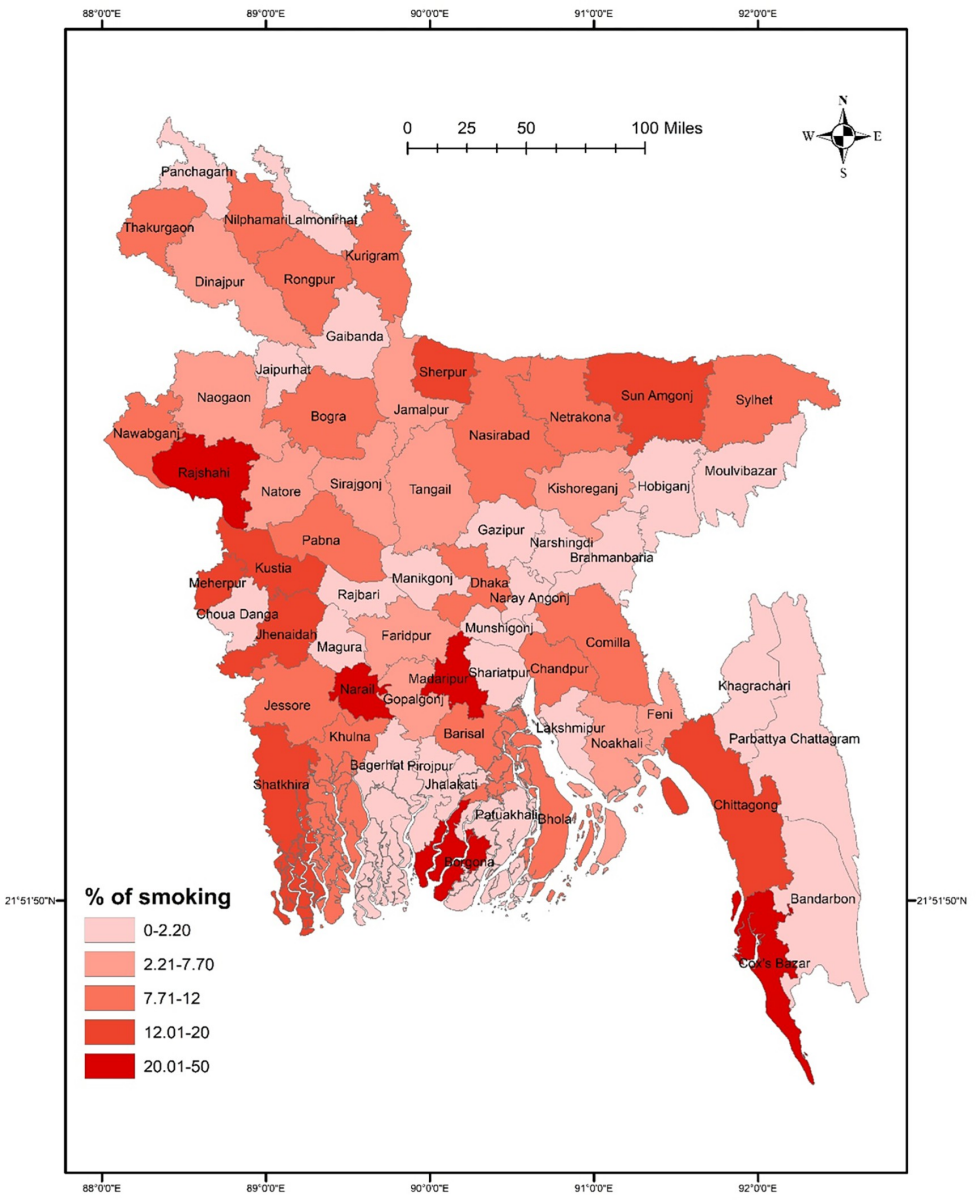
Prevalence of smoking among university entrance test-taking students in Bangladesh.

**Fig 2:**
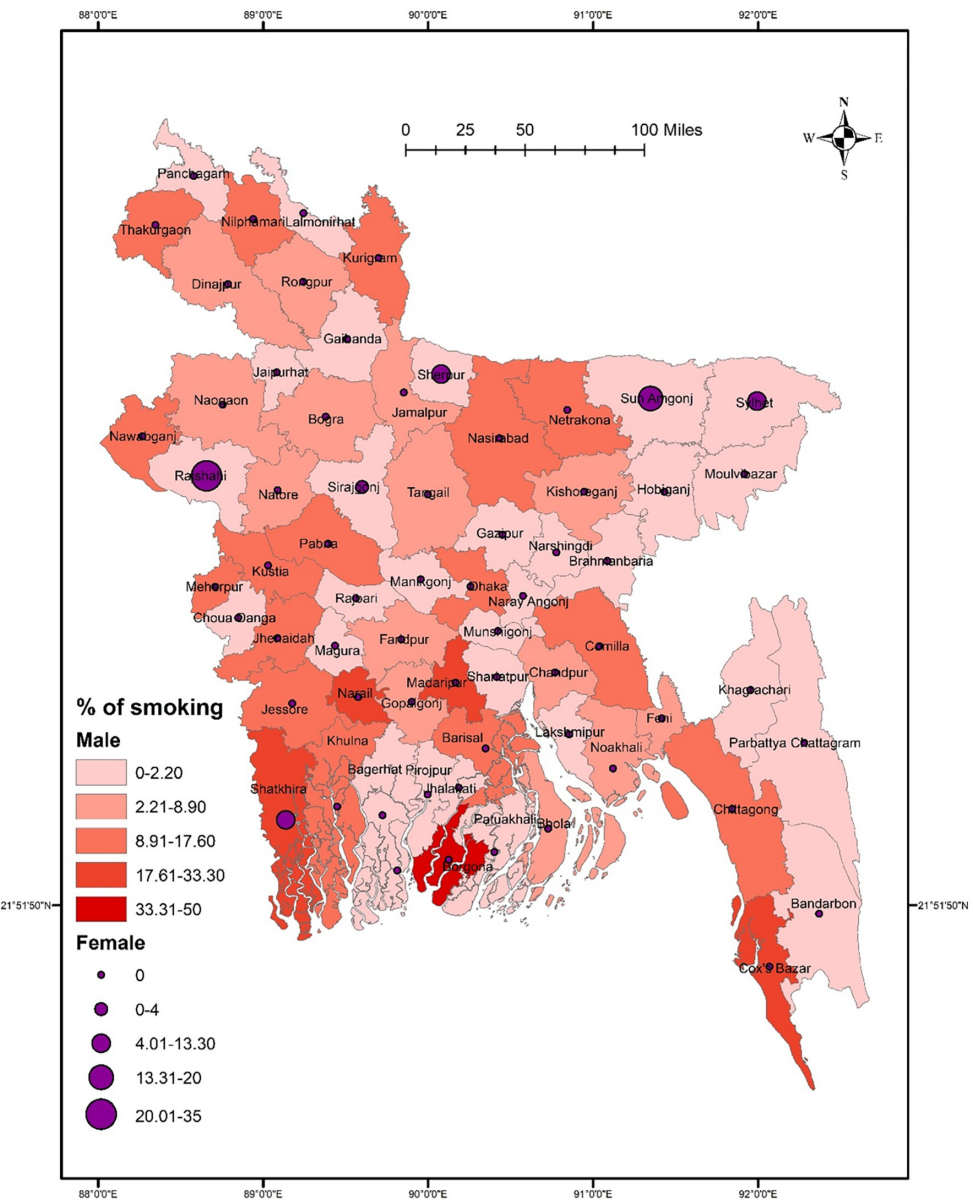
Prevalence of gender-based smoking among university entrance test-taking students in Bangladesh.

**Fig 3:**
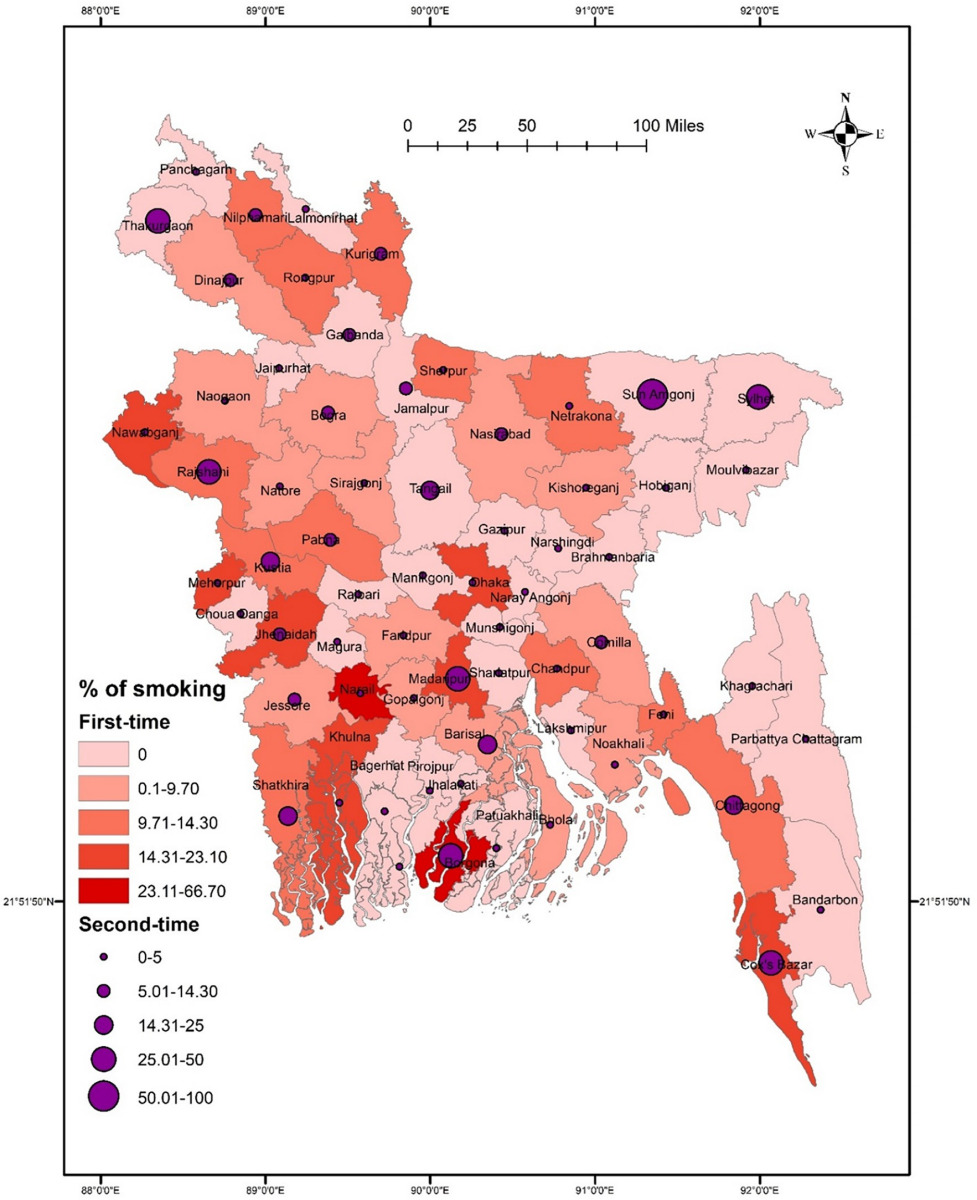
Prevalence of student status-based smoking among university entrance test-taking students in Bangladesh.

Results suggested no significant association between districts and smoking (χ^2^ = 74.928, *p* = 0.108). The prevalence of smoking was higher in Rajshahi, Narail, Madaripur, Borgona, and Cox’s Bazar than in other districts. However, a low smoking prevalence was reported in Narshingdi, Gazipur, Brahmanbaria, Hobiganj, and Moulvibazar (**Fig 1**). Smoking did not significantly vary in terms of sex (χ^2^ = 54.074, *p* = 0.587), though a higher rate of smoking was found among males in some northern districts such as Thakurgaon, Nilphamari, and Kurigram, and some southern districts such as Narail, Borgona, Madaripur, and Shatkhira (**Fig 2**). However, females had a significantly higher smoking rate in Sherpur, Sunamganj, Sylhet, and Rajbari than in other districts (**Fig 2**). In terms of student-status-based distribution, repeat test-taking students had a significant association between smoking and district location (χ^2^ = 70.212, *p* = 0.047). A higher smoking rate was detected in Thakurgaon, Sunamgonj, Sylhet, Rajshahi, Borgona, Madaripur, and Cox’s Bazar **(Fig 3).** However, first-time test-taking students had a higher prevalence of smoking in Nilphamari, Rangpur, Kurigram, Narail, Khulna, and Jhalokati (χ^2^ = 52.267, *p* = 0.780) than in other districts (**Fig 3**).

**Fig 4:**
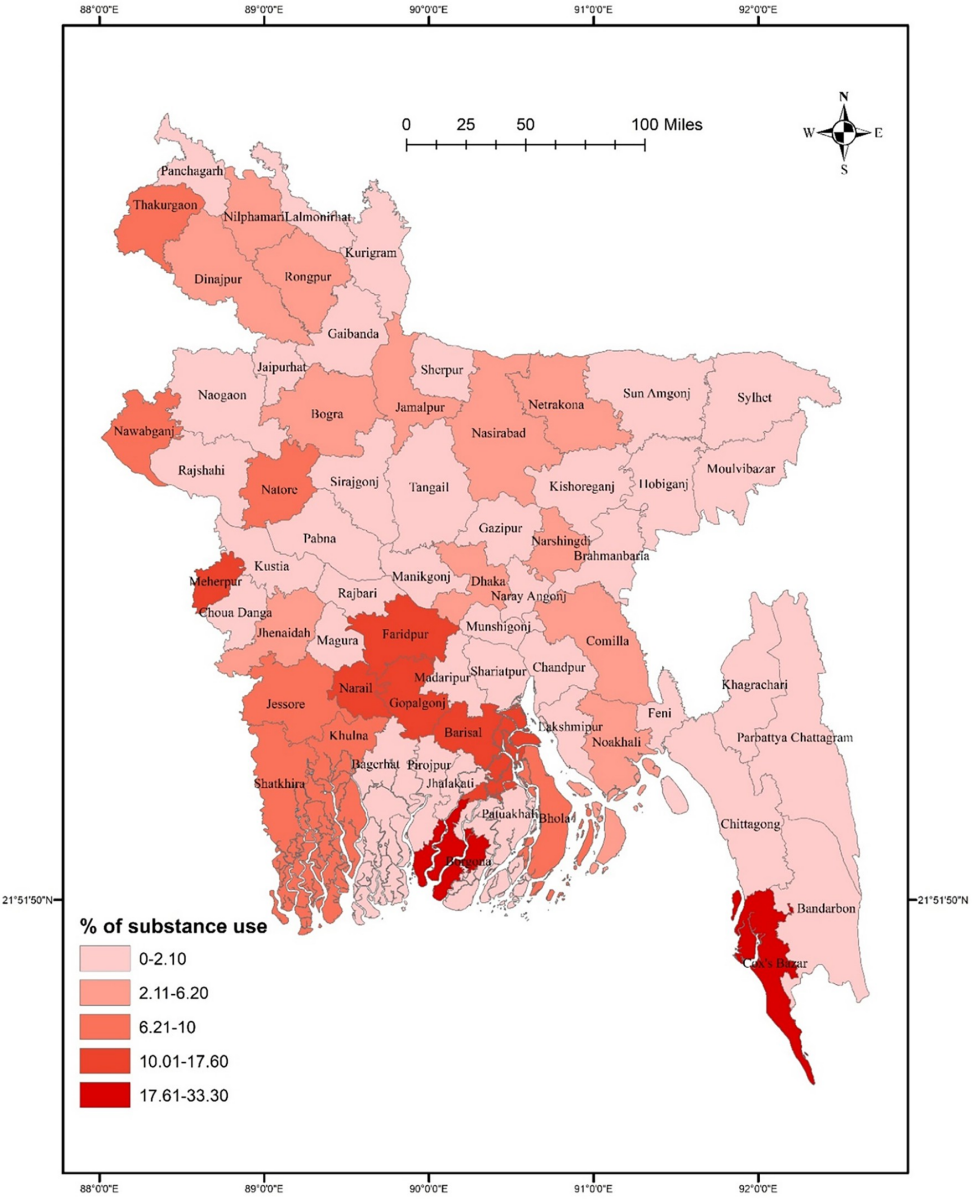
Prevalence of substance use among university entrance test-taking students in Bangladesh.

**Fig 5:**
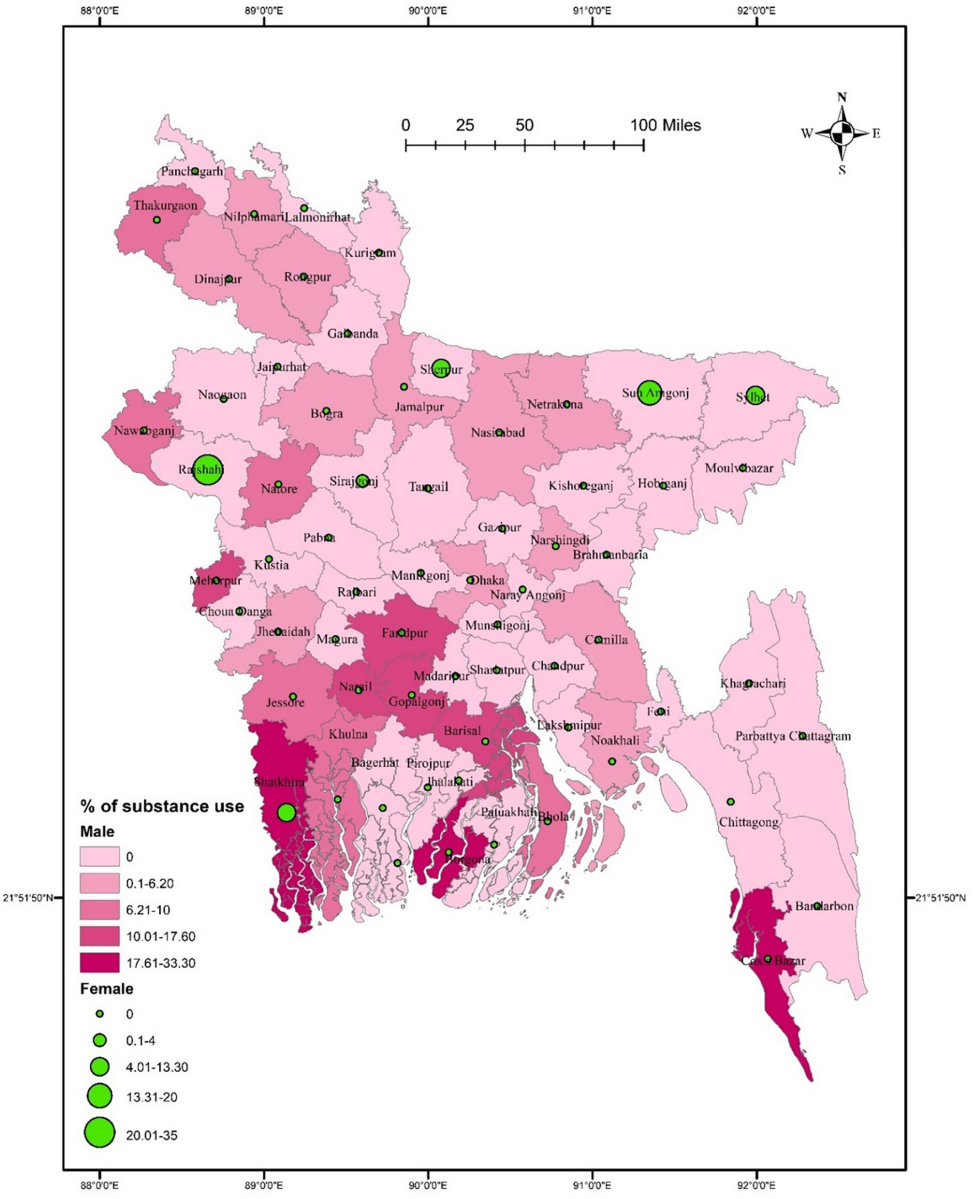
Prevalence of gender-based substance use among university entrance test-taking students in Bangladesh.

**Fig 6:**
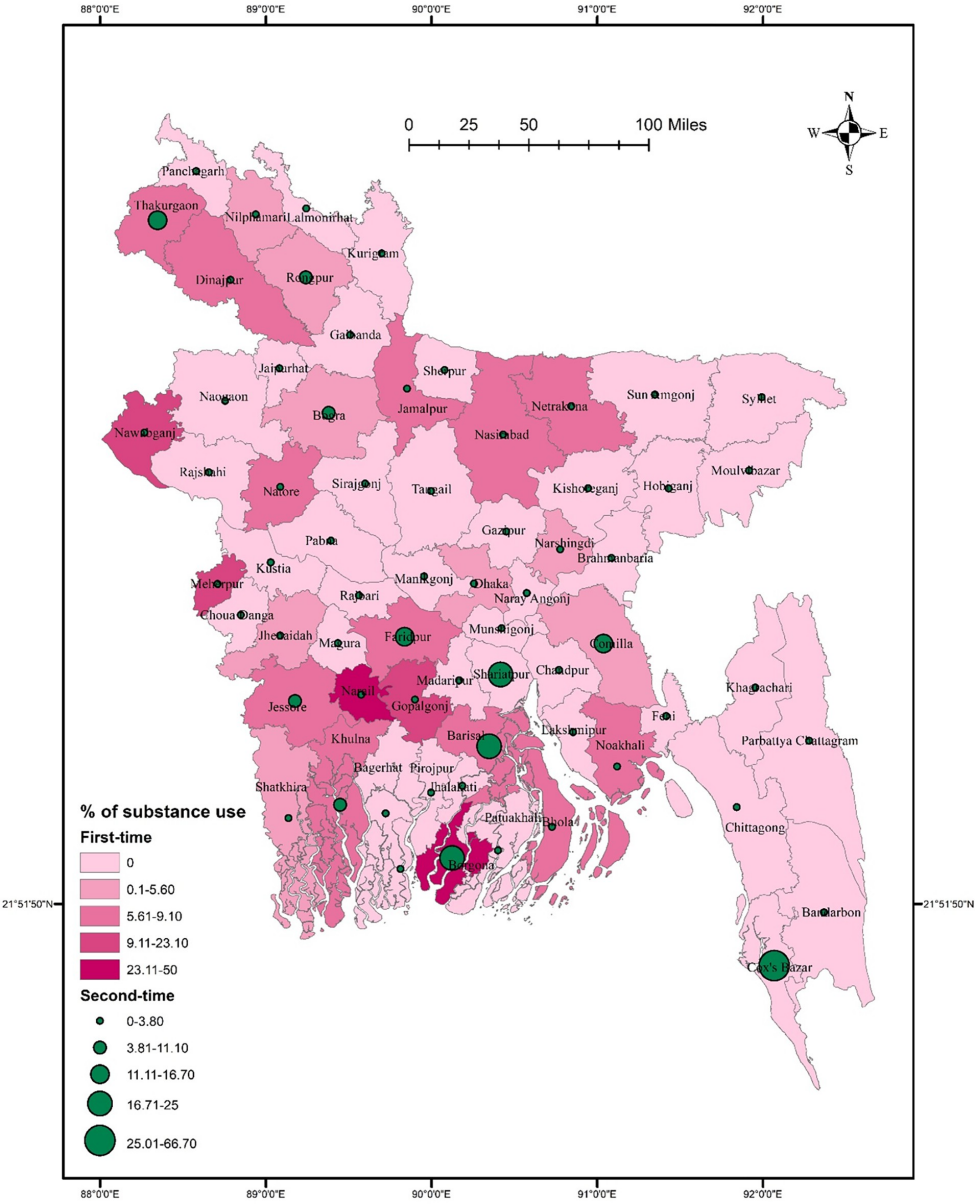
Prevalence of student status-based substance use among university entrance test-taking students in Bangladesh.

There was no significant association between district residence and substance use (χ^2^ = 78.968, *p* = 0.061). Substance use prevalence was higher in southern districts such as Faridpur, Narail, Gopalgonj, Barisal, Borgona, Khulna, and Cox’s Bazar than in other districts (**Fig 4**). Substance use did not significantly vary in terms of gender (χ^2^ = 75.173, *p* = 0.054 for males, and χ^2^ = 3.445, *p* = 0.969 for females). Males had a higher rate of substance use in some southern districts, such as Faridpur, Narail, Gopalgonj, Barisal, Borgona, Khulna, and Cox’s Bazar than in other districts. In contrast, females from Rajshahi, Sunamgonj, Sylhet, and Shatkhira had comparatively higher rates of substance use **(Fig 5**). In terms of student-status-based distribution, repeat test-taking students had a significant association between substance use and district residence (χ^2^ = 71.454, *p* = 0.038). A higher rate of substance use was detected in Thakurgaon, Faridpur, Shariatpur, Borgona, Barisal, and Cox’s Bazar **(Fig 6**). However, first-time test-taking students had a higher prevalence of substance use in Thakurgaon, Dinajpur, Jamampur, Netrakona, and Borgona (χ^2^ = 65.849, *p* = 0.313) than in other districts (**Fig 6**).

## 4 Discussion

This study aims to investigate the prevalence of cigarette smoking and substance use and attendant geographical variability among students taking university entrance tests in Bangladesh. This sample encompasses the age corresponding to the transition from adolescence to adulthood, an important period during which addictive behaviors tend to be initiated and consolidated. The prevalence of current tobacco smoking was 10.3%, whereas 3.9% of the respondents reported being current substance users. Urban residence, repeat-test-takers, anxiety, burnout, and suicidal behavior were both predictors of cigarette use and substance use. Besides, the study revealed that females had a 1.98 times higher risk of cigarette smoking, while the gender difference in substance use was not statistically significant.

Although these findings are consistent with previous studies in Bangladesh that surveyed a similar student population, our geolocation findings indicated that a substantial heterogeneity in the prevalence of such behaviors is present, thereby providing opportunities for more targeted interventions in regions in which the prevalence of substance use is particularly elevated. For instance, a study conducted among eleventh-grade high school students (on average one year younger than the current sample) reported very similar prevalence rates of tobacco smoking, drug use, and alcohol consumption, that is, 10.4%, 2.8%, and 3.1%, respectively [23]. Similarly, students from Iran reported that in the past 30 days, 9.3% smoked cigarettes and 9.3% smoked hookah, whereas the rate was 7% for illegal drugs and 9.5% for alcohol use at least once [24]. However, 62.4% of the participants from a university in Ethiopia reported using at least one substance, with alcohol being the most used (50.2%) among them [25]. Another study demonstrated that 70% used tobacco products, 79% used chat and 99% used alcoholic beverages [26].

Notably, a concerning trend has been observed in Bangladesh, where a substantial proportion of adolescents have been reported to initiate tobacco use at a remarkably young age with around 50% starting before the age of 10 years [27]. This early initiation of substance use poses significant health risks and underscores the importance of preventive measures targeting this vulnerable population. Furthermore, students transitioning from high school to university may encounter new environments and social pressures that can influence their substance use behaviors. In accordance with such concerns, we explored here a uniquely vulnerable and stressful period in the lives of high school graduates. That is, the process of preparing and then taking the highly competitive entrance examinations for acceptance into university studies. Unsurprisingly, during this highly stressful transitional period, it would be expected that increases in substance use behaviors would occur. Indeed, studies on university students in Bangladesh have reported higher prevalence rates of tobacco smoking, with figures as high as 36.1% [28] and even 60.2% [29] among specific cohorts. However, given the potential risks associated with this transitional period, future research must focus on understanding the factors that contribute to increased substance use among university students in Bangladesh. Exploring how these students are exposed to and influenced by substance use and identifying effective prevention and intervention strategies should be a priority, particularly when, as evidenced by our GIS data, particular regions emerge as highly vulnerable

A previous study reported that females are more likely to report higher risky behaviors such as tobacco smoking [30]. Concordant with such previous estimates, we found that female exam takers were twice more likely to smoke cigarettes, but that there was no significant difference between genders regarding substance use. However, a Portuguese study among adolescents reported a higher prevalence of frequent alcohol and tobacco use among boys rather than among girls (21.31% vs. 10.36%) [31], which is also consistent with other studies reporting less likely exposure to alcohol and marijuana among female genders [32]. These findings highlight the complex nature of gender disparities in unhealthy behaviors and the potential importance of cultural elements.

In the rural-urban context, variations in substance use among rural and urban students are closely tied to their grade levels. For example, rural middle school students tend to exhibit higher rates of alcohol, tobacco, and chewing tobacco use. In contrast, urban high school students show higher rates of illicit drug use [33]. Another study showed that rural individuals initially use less alcohol and marijuana than their urban peers during their freshman years but such differences disappear by their junior year, such that rural-urban divergences in the prevalence of tobacco use were not detected although the higher prevalence of tobacco use persisted among rural minorities [32]. Surprisingly, the present study shows a different trend, with urban residents at higher risk for both substance and tobacco use. The difference may be due to the less stringent imposition of traditional social norms and increased peer influence in urban areas. Urban students, often from higher-income families, are less financially burdened by smoking and substance use, consistent with the present study’s findings associating higher family income with a greater risk of risky behaviors (e.g., alcohol and marijuana use) as reported elsewhere [34].

In Bangladesh, gaining admission to a university for tertiary education is highly competitive, with limited seats available for a large number of high school graduates. The pressure of preparing for multiple exams and taking entrance tests at various locations can significantly impact student’s mental health. Those with underlying mental health issues are more likely to report substance use, as evidenced by the present study findings and other studies [35, 36]. The present study found that anxious test-taking students were at 1.56- and 2.48 times higher risk of tobacco smoking and substance use. Similarly, students with suicidal behaviors were 1.57 times and 2.76 times more likely to smoke cigarettes and report substance use. These findings align with previous research indicating a bidirectional relationship between mental health problems and substance use. For instance, two studies [36, 37] reported a high co-occurrence of substance use disorders with mood and anxiety disorders, whereas other studies highlighted the role of substances like cannabis as a coping mechanism for individuals with social anxiety [38]. Anxiety disorders can drive individuals to use substances as a form of self-medication to alleviate symptoms. This creates a vicious cycle, where substance use temporarily reduces anxiety but ultimately worsens mental health, increasing dependence and exacerbating both conditions [39]. Similarly, individuals with suicidal behavior may turn to substance use to escape emotional pain [40]. This maladaptive coping strategy significantly raises the risk of developing substance use disorders, further complicating mental health treatment and overall well-being.

The GIS distribution analysis conducted in this study revealed that, overall, there was no significant difference in the distribution of both smoking and substance use across various districts. However, it’s worth noting that certain districts, namely Rajshahi, Narail, Gopalgonj, Faridpur, Borgona, and Cox’s Bazar, reported higher prevalence rates of these behaviors. When examining the data further, *post-hoc analysis* revealed interesting gender-wise differences across the districts. Specifically, a significant difference in substance use was observed among males across the districts, whereas tobacco smoking exhibited significant differences among females. Moreover, the study also explored the association between the status of test-taking students and district-wise distribution of smoking and substance use. Intriguingly, repeat test-taking students showed a significant association with the district-wise distribution of both cigarette smoking and substance use. Indeed, districts such as Thakurgaon, Sunamgonj, Sylhet, Rajshahi, Borgona, Madaripur, and Cox’s Bazar reported higher prevalence rates of smoking, while Thakurgaon, Faridpur, Shariatpur, Borgona, Barisal, and Cox’s Bazar exhibited higher rates of substance use among repeat test-taking students. These district-wise patterns of smoking and substance use could be influenced by the country’s geographical location, making it easier for substances to cross the border from neighboring countries. Additionally, the lack of regional cooperation and limited capacity in law enforcement may contribute to the higher prevalence rates of smoking and substance use in these specific districts. However, it is important to emphasize that no significant association was found between the overall smoking and substance use status and specific districts, suggesting that, on the whole, there were no significant variations in prevalence rates across districts.

The present study has some limitations to be discussed. Firstly, the study’s cross-sectional design restricts the ability to establish causal relationships between cigarette and substance use and the variables of interest. Secondly, the non-probability sampling technique can encompass potential bias. Additionally, the study’s was focused on a single academic center which limits the external validity of the results, as factors specific to that center may have influenced the outcomes. Finally, self-reported data may include subjectivity and recall biases. Further study needs to consider this cohort with a more rigorous study design.

## 5 Recommendations

To address the issues of cigarette smoking and substance use among students taking university entrance tests, several targeted strategies are essential. These recommendations are designed to provide a holistic approach to improving student well-being and reducing substance use. These recommendations aim to create a supportive environment for students, helping them manage stress effectively and reducing the prevalence of substance use.

- Firstly, universities and educational institutions should establish robust mental health support programs to help students manage the stress and anxiety associated with university entrance exams. This could include counseling services, stress management workshops, and peer support groups.
- Secondly, integrating comprehensive substance use prevention education into the high school and university curricula can raise awareness about the risks of tobacco and substance use. These programs should provide students with practical strategies to cope with exam-related stress without resorting to harmful substances.
- Thirdly, encouraging students to adopt healthy lifestyle choices can reduce the likelihood of substance use. Institutions can offer programs that promote physical activity, healthy eating, and adequate sleep, which are all crucial for maintaining mental and physical health during stressful periods.
- Finally, early screening for anxiety, depression, and other mental health issues among students can lead to timely intervention. Schools and universities should implement regular mental health assessments and provide immediate support to students exhibiting signs of mental health problems or substance use.

## 6 Conclusions

In conclusion, this study provides valuable insights into the prevalence of smoking and substance use among students undergoing university entrance examinations in Bangladesh, a critical period in their transition from adolescence to adulthood. The findings highlight noteworthy trends, including early initiation of substance use, gender-based disparities, and geographical variations. Furthermore, the intricate relationships between mental health issues, notably anxiety and suicidal tendencies, and substance use emerged, which suggests the urgency of implementing preventive measures, targeted interventions, and a comprehensive approach encompassing mental health and substance use concerns to better support this transitioning population and promote their overall well-being during a time of heightened stress and uncertainty.

## Supporting information

S1 Dataset (SAV)
